# Exploiting correlated molecular-dynamics networks to counteract enzyme activity–stability trade-off

**DOI:** 10.1073/pnas.1812204115

**Published:** 2018-12-10

**Authors:** Haoran Yu, Paul A. Dalby

**Affiliations:** ^a^Department of Biochemical Engineering, University College London, WC1H 0AH London, United Kingdom

**Keywords:** dynamics, trade-off, protein engineering, transketolase, stability

## Abstract

The directed evolution of enzymes for improved activity or substrate specificity commonly leads to a trade-off in stability. We have identified an activity–stability trade-off and a loss in unfolding cooperativity for a variant (3M) of *Escherichia coli* transketolase (TK) engineered to accept aromatic substrates. Molecular dynamics simulations of 3M revealed increased flexibility in several interconnected active-site regions that also form part of the dimer interface. Mutating the newly flexible active-site residues to regain stability risked losing the new activity. We hypothesized that stabilizing mutations could be targeted to residues outside of the active site, whose dynamics were correlated with the newly flexible active-site residues. We previously stabilized WT TK by targeting mutations to highly flexible regions. These regions were much less flexible in 3M and would not have been selected a priori as targets using the same strategy based on flexibility alone. However, their dynamics were highly correlated with the newly flexible active-site regions of 3M. Introducing the previous mutations into 3M reestablished the WT level of stability and unfolding cooperativity, giving a 10.8-fold improved half-life at 55 °C, and increased midpoint and aggregation onset temperatures by 3 °C and 4.3 °C, respectively. Even the activity toward aromatic aldehydes increased up to threefold. Molecular dynamics simulations confirmed that the mutations rigidified the active-site via the correlated network. This work provides insights into the impact of rigidifying mutations within highly correlated dynamic networks that could also be useful for developing improved computational protein engineering strategies.

## Significance

Rigidifying flexible sites is a powerful method to improve enzyme stability. However, if the highly flexible regions form the active site, modifying them risks losing activity due to the activity–stability trade-off. We hypothesized here that regions outside the active site whose dynamics were highly correlated to flexible active sites, would provide good targets for stabilizing mutations. To test this hypothesis, six variants were constructed in the 3M variant of *Escherichia coli* transketolase. The best variant had a 10.8-fold improved half-life at 55 °C, and increased the *T*_m_ and *T*_agg_ by 3 °C and 4.3 °C, respectively. The variants even increased the activity, by up to threefold. This study highlights how protein engineering strategies could be potentially improved by considering long-range dynamics.

Enzymes are natural catalysts with great potential for industrial applications. However, the natural substrate specificity of enzymes often fails to meet the requirement of industrial chemists. Directed evolution has been a powerful tool to obtain enzyme variants with expanded substrate scope. However, during the process of directed evolution for improved activity or substrate specificity, stability loss is commonly observed when mutations are accumulated primarily for function (1, 2). This negative correlation between enzyme stability and activity, the so-called activity–stability trade-off, has been well documented (3–5). From the point of view of overall protein stability, the active-site organization is already inherently unfavorable as functional residues—generally polar or charged—are embedded in hydrophobic clefts, sometimes with proximal like-charges (6, 7). While natural enzymes have evolved extensively throughout their entire sequence, to maintain a delicate balance between function and stability the introduction of a sparse number of function-modifying mutations in a few rounds of directed evolution is highly likely to decrease their stability.

*Escherichia coli* transketolase (TK) is a thiamine diphosphate (ThDP) and Mg^2+^-dependent homodimeric enzyme that catalyzes the reversible transfer of a C2-ketol unit from d-xylulose-5-phosphate to either d-ribose-5-phosphate or d-erythrose-4-phosphate, linking glycolysis to the pentose phosphate pathway (8). The stereospecifically controlled carbon–carbon bond-forming ability makes it promising as a biocatalyst in industry for the synthesis of complex carbohydrates and other high-value compounds (9). To increase potential use for the synthesis of novel dihydroxy-ketone compounds, *E. coli* TK has been engineered by a series of smart-library approaches to expand its substrate scope from phosphorylated to nonphosphorylated polar acceptors, then to nonpolar aliphatic substrates, and on to hetero-aromatic and nonpolar aromatic substrates, which makes it a great model for investigation of the relationship between stability and new functions, when using guided or semirational-directed evolution strategies.

Saturation mutagenesis was first targeted independently to 10 active-site residues in contact with cofactor or substrate, and also to the 10 least-conserved second-shell residues (10). Various single mutants gave improved activity toward glycolaldehyde (GA), or propionaldehyde (PA), with either enhanced or reversed enantioselectivity (10–12). However, a trade-off was found between soluble expression levels and specific activity for all single mutants (13). The loss of soluble expression levels in cells could be linked to the loss of stability (14). For example, a single mutant, D469T, showing fivefold increased specific activity toward PA, gave 53% of the soluble expression level observed for WT, and decreased the temperature at which aggregation was induced (*T*_agg_) by 11 °C, reflecting a typical activity–stability trade-off (13). A statistical coupling analysis (SCA) approach was applied to target networks for restabilization, and a solubly expressed double-variant D469T/R520Q was identified with 9.6-fold improved specific activity relative to WT (13).

Later, saturation mutagenesis of two TK active-site residues within D469T/R520Q led to several variants, including S385Y/D469T/R520Q (3M), that were active on three benzaldehyde derivatives, in contrast to WT TK, which was active only on nonaromatic aldehydes (15, 16). Interestingly, the kinetic analysis of 3M showed that this variant improved the activities toward the three benzaldehyde analogs, 3-formylbenzoic acid (3-FBA), 4-formylbenzoic acid (4-FBA), and 3-hydroxybenzaldehyde (3-HBA), in three different ways. The crystal structure of 3M revealed that the directed-evolution had generated an evolutionary intermediate with divergent binding modes for the three aromatic aldehydes tested (17).

The 3M variant is promising for the biocatalytic synthesis of novel aromatic dihydroxyketones and subsequent transamination to access aromatic aminodiols, such as chloramphenicol analogs. A future aim should be to further engineer the function of this variant. However, such a strategy would be most successful when starting from an enzyme with a stability that is at least comparable to the WT. Here we have measured the thermal stability of the 3M variant and identified a clear activity–stability trade-off linked to a loss in unfolding cooperativity.

The possible origin of this trade-off was initially investigated using molecular dynamics (MD) simulations. It was found that regions around the active-site mutations (S385Y/D469T/R520Q) in 3M, and other nearby residues, became more flexible at high temperature and led to the decrease in stability and unfolding cooperativity. These regions were clustered in the part of the dimer interface that forms the two identical active sites. However, active-site residues do not represent a good target for directed evolution, and less so for rational design, due to the risk of compromising the newly gained activity. To restore this loss of stability within the 3M variant, we hypothesized that those regions outside the active site but correlated by their dynamics to active-site flexibility, would be good targets for stabilizing mutations. Four mutations—including H192P, A282P, I365L, and G506A—were known to stabilize the WT *E. coli* TK (18–20), and were now found to be located in regions whose dynamics correlated with those of the flexible active sites in 3M. Six variants of 3M were constructed and characterized, of which four increased the thermostability, and yet retained or improved up to threefold, the enzyme kinetic parameters for aromatic aldehyde substrates.

## Results and Discussion

### Investigation of the Activity–Stability Trade-Off in TK.

The variant S385Y/D469T/R520Q (designated 3M) was obtained previously by directed evolution toward aromatic aldehyde substrates (16). The specific activities for 3M and WT, toward 50 mM of 3-FBA, 4-FBA, or 3-HBA determined previously (16), and also in this work toward 50 mM GA or PA, are summarized in Table 1. Interestingly, the 3M variant retained activity on the aliphatic aldehydes GA and PA, with specific activities of 0.75 µmol·mg^−1^·min^−1^ and 1.85 µmol·mg^−1^·min^−1^, respectively. Thus, directed evolution toward 3M not only shifted the substrate specificity toward previously unaccepted aromatic aldehydes, but also increased its promiscuity compared with WT TK.

**Table 1.** Promiscuity of TK variants

Variant	Specific activity (µmol·mg^−1^·min^−1^)				
	GA	PA	3-FBA*	4-FBA*	3-HBA*
WT	48.8 (0.3)	0.029 (0.001)	0	0	0
3M	0.75 (0.001)	1.85 (0.01)	2.31	1.53	0.088
5M	0.88 (0.08)	0.92 (0.03)	3.03 (0.51)	1.17 (0.03)	0.29 (0.005)
7M	1.14 (0.09)	1.90 (0.24)	2.36 (0.57)	3.01 (0.44)	0.19 (0.01)

* Specific activity data for WT and 3M was from ref. 16. The specific activities were measured in 50 mM Tris⋅HCl, 2.4 mM ThDP, 9 mM MgCl_2_, pH 7.0, toward 50 mM 3-FBA/50 mM HPA, 30 mM 4-FBA/50 mM HPA, and 15 mM 3-HBA/30 mM HPA, respectively. 3M, S385Y/D469T/R520Q; 5M, H192P/A282P/S385Y/D469T/R520Q; 7M, H192P/A282P/I365L/S385Y/D469T/G506A/R520Q.

Thermal transition midpoint temperatures, *T*_m_, were determined from intrinsic fluorescence measurements on WT and the variant S385Y/D469T/R520Q for comparison (*SI Appendix*, Fig. S1*A*). Their *T*_agg_ were simultaneously determined from static light-scattering (SLS) measurements (*SI Appendix*, Fig. S1*B*). The *T*_m_ and *T*_agg_ values of the 3M variant were 2.3 °C and 3.1 °C lower, respectively, than those of WT (Table 2), suggesting that the variant had achieved new functions at the cost of a significant trade-off in thermal stability.

**Table 2.** Characteristics of variant TKs

Variant	*T*_m_ (°C)	*∆S*_*vh*_	*f*_*55*_	*T*_agg_ (°C)	*k*_d_ × 10^3^ (%^−1^ min^−1^)	Half-life *t*_1/2_ (min)*	*K*_m_ (mM)^†^	*k*_*cat*_ (s^−1^)	*k*_cat_/*K*_m_ (s^−1^ M^−1^)
WT	65.4 (0.08)	0.27 (0.04)	0.014 (0.006)	62.9 (0.5)	ND	ND^‡^	ND	ND	MD
3M	62.9 (0.4)	0.18 (0.003)	0.091 (0.005)	59.8 (0.3)	1.65 (0.19)	6.1(0.4)	2.3 (0.3)	2.8 (0.1)	1,217
3M/H192P	63.4 (0.4)	0.24 (0.02)	0.046 (0.003)	60.1 (0.4)	ND	ND	2.2 (0.4)	3.5 (0.1)	1,591
3M/A282P	63.5 (0.5)	0.18 (0.01)	0.09 (0.01)	61.0 (0.7)	ND	ND	2.1 (0.2)	3.2 (0.3)	1,524
5M	62.8 (0.2)	0.20 (0.05)	0.044 (0.006)	61.4 (0.7)	0.28 (0.03)	36 (2)	1.3 (0.2)	2.9 (0.1)	2,231
5M/I365L	64.4 (0.1)	0.22 (0.1)	0.053 (0.002)	64.0 (0.6)	0.098 (0.01)	103 (6)	1.3 (0.4)	2.1 (0.3)	1,615
5M/G506A	64.2 (0.3)	0.30 (0.08)	0.034 (0.006)	61.8 (0.2)	0.17 (0.02)	60 (4)	2.7 (0.4)	3.9 (0.2)	1,444
7M	65.8 (0.2)	0.25 (0.05)	0.023 (0.009)	64.1 (0.5)	0.15 (0.02)	66 (4)	2.3 (0.3)	3.8 (0.3)	1,652

* Half-life at 55 °C, in 50 mM Tris⋅HCl, pH 7.0, 2.4 mM ThDP, 9 mM Mg^2+^.

^†^ Enzyme kinetic parameters for 3-FBA at 22 °C, 50 mM HPA, 50 mM Tris⋅HCl, pH 7.0, 2.4 mM ThDP, 9 mM Mg^2+^.

^‡^ Half-life of WT was 4.0 ± 0.3 min at 60 °C, in 50 mM Tris⋅HCl, pH 7.0, 2.4 mM ThDP, 9 mM Mg^2+^.

### MD Simulations to Explore the Origin of Activity–Stability Trade-Off.

#### Variant 3M became more flexible.

**Fig. 1.**
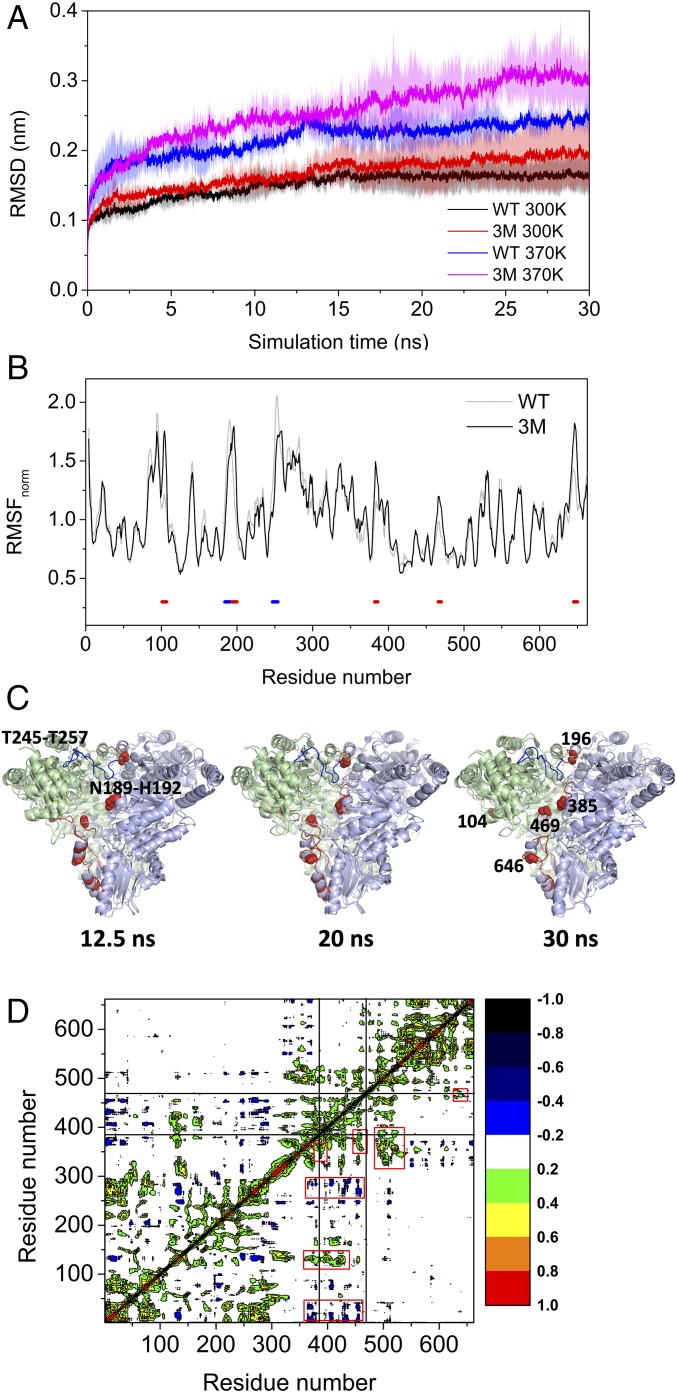
MD simulation analysis of 3M variant. (*A*) Comparison of RMSD values of WT and the 3M variant calculated from triplicate 30-ns MD simulations at 300 K and 370 K. (*B*) Comparison of normalized RMSF of WT and the 3M variant. Red, residues with ΔRMSF between variant and WT > 0.2; blue, residues with ΔRMSF < −0.2. (*C*) Structural alignment between WT and 3M at three key simulation timepoints. Light green, chain A; light blue, chain B; red, flexible regions in the 3M including G99-Y105, G195-D200, D381-N386, L466-G470, V630-K660; blue, regions stabilized in the 3M including N185-H192 and T245-T257. The five flexible residues in the 3M are shown as spheres. (*D*) DCCM for the 3M variant, based on the last 10-ns of the MD simulation trajectory at 370 K, and averaged from triplicate runs.

MD simulations were applied to investigate the flexibility changes of the 3M variant structure at 300 K and 370 K. RMSD values calculated relative to the starting structure for the backbones of all residues were used to explore the overall conformational flexibility of the native structure (Fig. 1*A*). The RMSD values calculated from MD simulations were higher at 370 K than at 300 K, reflecting that MD simulation could effectively depict the thermal motion information of the structure. MD simulations were run for 30 ns and during most of that time, the RMSD values of the 3M variant were higher than those of WT, at both 300 K and 370 K, indicating increased conformational flexibility in the 3M variant, which would have contributed to the decreased stability of 3M. The differences between the RMSD values of 3M and WT were higher at 370 K than at 300 K, implying that the higher temperature provided a more sensitive probe of the impact of the mutations on the protein flexibility, and could also potentially give more insight into the onset of protein unfolding (Fig. 1*A*).

#### Variant 3M destabilized local structures at the dimer interface.

The local flexibilities of 3M were further analyzed from the normalized root mean square fluctuation (RMSF) values at 370 K, as previously described (19) (Fig. 1*B*). Compared with WT, 3M had higher flexibility (red zones in Fig. 1*B*)—predominantly within five local regions around the mutation sites S385Y and D469T, and also around Gly104, Trp196, and Glu646—that were all colocated at the interface between the two TK monomers (Fig. 1*C*). This dimer interface network was connected end-to-end in a continuous stripe across the protein. The structural alignment between WT and 3M indicated that these flexible regions in 3M underwent significant local unfolding during the simulation at 370 K (Fig. 1*C*). For example, the region around Glu646 was α-helical at 12.5 ns for both the WT and 3M. However, this helix was lost at 20 and 30 ns in 3M, but remained structured in WT (Fig. 1*C*). Similar local unfolding was also found for the region around Ser/Tyr385 in 3M, while significant movement in the positions of Gly104 and Thr469 could also be observed between the different time points for 3M, compared with WT (Fig. 1*C*). This increased flexibility and local unfolding at the dimer interface in 3M would have weakened the interactions between monomers and resulted in destabilization of the whole structure.

The five flexible regions identified in 3M formed a structural interaction network, and the increased flexibility in 3M appears to have resulted from a net removal of interactions across the network (*SI Appendix*, Fig. S2). For example, the D469T mutation removed two salt bridges with Arg91 and His100, and replaced them with weaker hydrogen bond interactions. D469T also removed two hydrogen bonds to Gly99 and His26 (*SI Appendix*, Fig. S2). R520Q removed hydrogen bonds to Glu468, Leu466, and Gly465, which removed the direct connection with Thr469 (*SI Appendix*, Fig. S2). S385Y removed a hydrogen bond to Ala383 but established a new van der Waals contact with Ile189, and a new hydrogen bond with Gly262 (*SI Appendix*, Fig. S2).

The three active-site mutations—S385Y, D469T, and R520Q—that formed 3M from WT, also induced a decrease in flexibility at two sites, spanning N185-H192 and T245-T257 (blue zones in Fig. 1*B*). The fragment N185-H192 is also located in the dimer interface and formed several hydrogen bonds, including Ser188-Asp381, Ile189-Asp381, Asp190-Asp381, and Asp190-His406 between the two monomers in both the WT and 3M, whereas the fragment of T245-T257 does not form any interactions across the dimer interface (Fig. 1*C*). Interestingly, these two regions were identified previously as highly flexible surface loops in WT, and were consequently selected as mutation targets for engineering thermostability (19). Stabilization of these two fragments in 3M thus may have at least partially offset the destabilizing effect of the five flexible regions toward protein unfolding at high temperatures.

### Engineering 3M for Improved Stability.

The decreased stability of S385Y/D469T/R520Q (3M) could hamper the design of new variants aimed at improving the bioconversions with aromatic aldehydes, but also constrains the use of 3M at elevated temperatures that enhance the solubility of aromatic substrates in water. The strategy of rigidifying flexible sites (RFS) has been proven to be a powerful method to improve the stability of enzymes (21). Using both the RFS and also a consensus mutation approach, several variants of WT TK were found previously to be more thermostable (18–20, 22). One option might be to explore the WT-stabilizing mutations within 3M. However, the flexible regions that were originally targeted for stabilization by RFS in WT would not a priori appear to be good targets for stabilization of the 3M variant, when using our previous strategy that was based solely on ranking the flexibility of residues. For example, the highly flexible region W279-I290 in WT was stabilized previously with the A282P mutation (19), but this region was no longer highly flexible in the 3M variant (Fig. 1*B*). Similarly, the flexible region N185-H192 in WT was the target for the H192P mutation, but this region was also less flexible in 3M. Even the highly flexible loop T245-T257, targeted in WT but without yielding any stabilizing mutations (19), was already significantly rigidified in 3M.

To engineer stability of 3M, a better option might be to directly rigidify the flexible regions identified by MD simulations (Fig. 1*B*). However, these regions also formed the active site of the 3M variant, and involved the mutations that introduced the new activity toward aromatic aldehydes. Modifying them risked the loss of this activity in a reverse of the stability–activity trade-off.

We previously found that the dynamics underpinning the flexibility of different regions of TK were highly correlated across long distances (22). Specifically, a mutation at one site could influence the dynamics of regions far away from it. Additionally, we had also previously used SCA to identify evolutionarily correlated networks of residues that included the active-site residues we had already mutated for function. Screening of libraries targeted to one of these networks led to the R520Q mutation that stabilized TK variant D469T sufficiently to restore soluble expression (13) and enable the subsequent active-site mutation S385Y to form variant 3M (16). Inspired by these findings, we hypothesized here that regions outside the active site, but whose dynamics were highly correlated to the flexible regions within the active site at the dimer interface, would provide good targets for stabilizing mutations, even if those target sites were not themselves highly flexible.

To identify the regions dynamically correlated with the flexible active-site regions in 3M, we computed the dynamics cross-correlation map (DCCM) for 3M. The regions undergoing flexibility change (Fig. 1*B*) also showed good dynamics correlations with each other (Fig. 1*D*). For example, the region around Tyr385 maintained a strong positive correlation with the region around Thr469, whereas Thr469 displayed a positive correlation with the region around Glu646 in the C terminal (Fig. 1*D*). Additionally, the regions around Tyr385 and Thr469 also showed negative (anti-)correlation with the dynamics in fragments across residues 250–300 that accounted for the stabilization of regions T245-T257 and W279-I290 in 3M.

The dynamics of other regions were also strongly correlated with those of the highly flexible 3M active-site/dimer-interface regions centered around Tyr385 and Thr469. These included anticorrelation with fragment 1–50, and positive dynamics correlation with the regions around residues 125, 360, and 500. Each of these regions retained a similar overall degree of flexibility between 3M and WT, and so only the direction and not the amplitude of these motions became correlated to those in the 3M active-site/dimer-interface.

Interestingly, some of the mutations that stabilized WT TK previously were located in the regions that had highly correlated dynamics with the highly flexible active-site/dimer-interface regions of 3M. For example, the mutation H192P is located in the fragment N185-H192, which had decreased flexibility in 3M, and had direct interactions with the region around Tyr385 (Fig. 1*C*). Similarly, the mutation A282P is located in the region 250–300, which was significantly less flexible in 3M compared with WT, but nevertheless had dynamics that were anticorrelated with those in regions around Tyr385 or Thr469. Finally, the mutations I365L and G506A are located in regions that had positive dynamics correlations with the flexible active-site regions in 3M. Therefore, we introduced these mutations into the 3M variant to examine whether they would also stabilize 3M via the correlated-dynamics network.

### Residual Activities of TK Mutants After Heating.

**Fig. 2.**
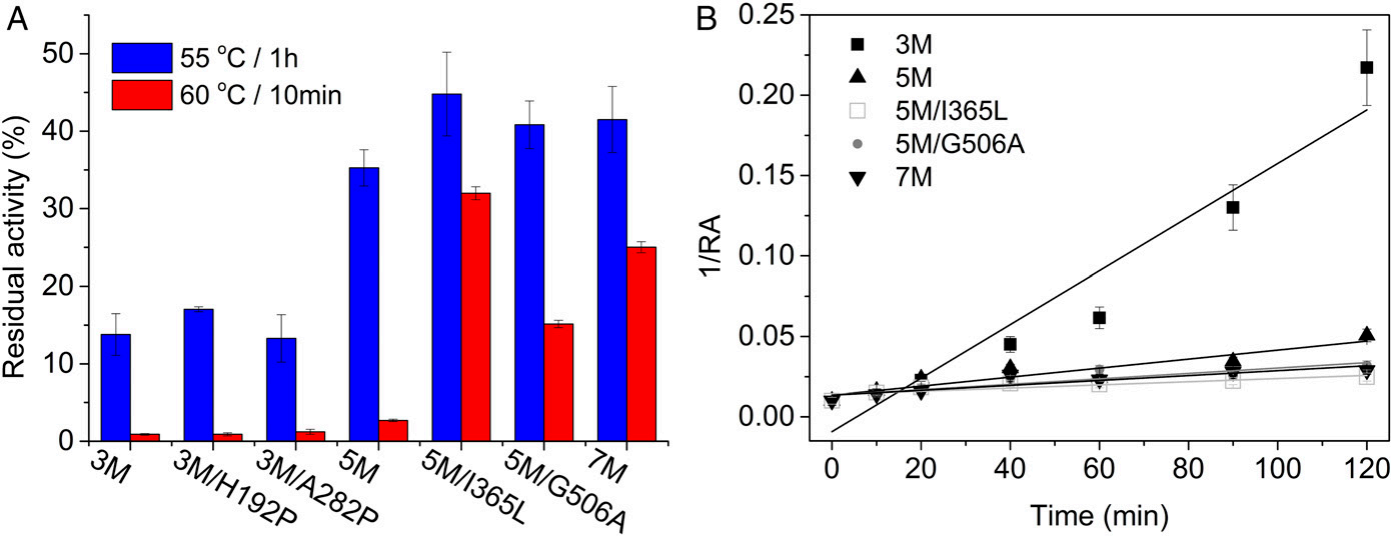
Thermal stability of TK variants. (*A*) The residual activity of TK variants. (*B*) Second-order kinetics of activity loss for TK variants at 55 °C.

Six new variants—3M/H192P, 3M/A282P, 3M/H192P/A282P (5M), 5M/I365L, 5M/G506A, and 5M/I365L/G506A (7M)—were constructed. As in previous work (18, 19), we evaluated the thermostability of the TK variants from their residual activity after temporarily heating at an elevated temperature. Of the six new variants, 5M, 5M/I365L, 5M/G506A, and 7M showed increased residual activity after heating at 55 °C for 1 h, whereas the initial two variants, 3M/H192P and 3M/A282P, each retained a similar activity to 3M after heating. When heated at 60 °C for 10 min, the variant 3M retained only 0.9% activity (Fig. 2*A*) and again variants 3M/H192P and 3M/A282P retained a similar level of activity to 3M. Interestingly, 5M also only retained 2.7% of the initial activity, suggesting that 60 °C was leading to more extensive thermal unfolding-induced aggregation of 5M. In contrast, 5M/I365L showed the highest residual activity of 32%, nearly 35-fold higher than that of 3M after heating at 60 °C. Two other variants, 5M/G506A and 7M, also displayed, respectively, 17- and 27.8-fold improvements in residual activity compared with 3M, after heating at 60 °C (Fig. 2*A*).

### Measurement of Half-Life for Stable Variants.

The half-life for the loss of enzyme activity at 55 °C was also determined for the four variants showing the highest residual activities relative to 3M, including 5M, 5M/I365L, 5M/G506A, and 7M. The 3M variant lost activity rapidly at 55 °C and maintained only 4.6% of the initial activity after 120 min, whereas 5M, 5M/I365L, 5M/G506A, and 7M had residual activities of 19.8%, 40.7%, 31.4%, and 34.8%, respectively (*SI Appendix*, Fig. S3). The degradation rate constant, *k*_d_, was calculated by fitting the denaturation data to a second-order degradation function (Fig. 2*B*). All four of the new variants had a lower *k*_d_ than 3M, indicating that the new variants deactivated more slowly. 5M/I365L had the longest half-life of 102.5 min, a 17-fold improvement over that for the 3M variant. The variants 5M, 5M/G506A, and 7M also had 5.9-, 9.8-, and 10.8-fold improved half-lives above that of 3M, with values of 35.8, 59.9, and 65.8 min, respectively (Table 2).

### Thermodynamic Stability of TK Variants.

The *T*_m_ and *T*_agg_ were also measured for the new variants. The *T*_m_ values for variants 3M/H192P, 3M/A282P, and 5M were essentially unchanged from that of the 3M variant, whereas 5M/I365L, 5M/G506A, and 7M increased the *T*_m_ over that of 3M, by 1.5 °C, 1.3 °C, and 2.9 °C, respectively (Table 2). It was interesting to find that the *T*_m_ of the variant 7M was even 0.6 °C higher than that of WT, indicating the successful reversal of the thermal stability lost in the 3M variant (Table 2). The increased *T*_m_ values of the variants were consistent with the improved residual activities observed after incubation for 10 min at 60 °C.

The thermodynamic stability, as measured indirectly by *T*_m_, and more specifically by the fraction unfolded at 55 °C, *f*_55_, can reveal the extent to which global unfolding is important for inactivation by aggregation. 5M/I365L had the longest half-life but its *T*_m_ was not the highest among the variants constructed. We therefore examined the linear correlations between *T*_m_ or *f*_55_ and the second-order degradation rate constant at 55 °C (Table 2 and *SI Appendix*, Fig. S4). Both *T*_m_ and *f*_55_ indicated potential correlations but with 5M/I365L as an outlier, indicating that global unfolding had a significant influence on the relative inactivation rates of the variants 55 °C. For all variants, *f*_55_ > 0.02 (2%), which is just above the 1% threshold observed by us previously for other proteins, as the point at which the role of global unfolding in aggregation kinetics becomes relatively insignificant compared with local fluctuations in the native ensemble (23–25).

Consistent with its *T*_m_, variant 7M had the highest *T*_agg_ value of 64.1 °C, which was 4.3 °C higher than that of 3M, and 1.2 °C higher than that of WT, indicating an increased tolerance to thermally induced aggregation (Table 2 and *SI Appendix*, Fig. S5). 5M and 5M/G506A also increased the *T*_agg_ by 1.6 °C and 2 °C, while those of 3M/H192P and 3M/A282P were essentially unchanged from that of the variant 3M (Table 2). 5M/I365L gave a dramatically different aggregation profile to all of the other variants (*SI Appendix*, Fig. S5). Its SLS signal increased slowly from 50 °C, but then more rapidly at above 64 °C. This behavior may be linked to its improved kinetic stability, as indicated by its residual activity and half-life measurements, but the mechanism is not clear. A more detailed discussion on this aspect can be found in *SI Appendix*.

### Kinetic Studies of TK Variant Activity.

Michaelis–Menten kinetics at saturating (50 mM) Li-hydroxypyruvate (Li-HPA), and varying concentrations of 3-FBA, were carried out to determine whether the mutations affected the enzyme kinetics at 22 °C. All new variants maintained comparable *K*_m_ and *k*_cat_ to the variant 3M, although 5M yielded an 80% increase in *k*_cat_/*K*_m_ (mainly due to *K*_m_). Thus, the inactivity toward aromatic aldehydes shown by WT was not reestablished when restoring thermostability into 3M, via mutations at sites whose dynamics correlated with those in the flexible 3M active-site (Table 2). The overall impact on promiscuity was also evaluated for 5M and 7M, for comparison with 3M and WT (Table 1). Interestingly, the variants 3M, 5M, and 7M had different effects on each of the substrates, with stabilization often also leading to increased specific activity.

For GA, the specific activity increased slightly from 3M to 5M, and also to 7M, giving a 50% increase overall. For PA, the specific activity decreased 50% from 3M to 5M, but increased back to the same level of 3M in 7M. For 3-FBA, the specific activity increased 30% from 3M to 5M and then decreased again in 7M. For 4-FBA, the specific activity decreased from 3M to 5M, but then increased in 7M such that it was double that of 3M. Finally, for 3-HBA, the specific activities of 5M and 7M were 3.3-fold and twofold higher than for 3M, respectively.

### Comparison of the Effects of Mutations on WT and 3M.

**Fig. 3.**
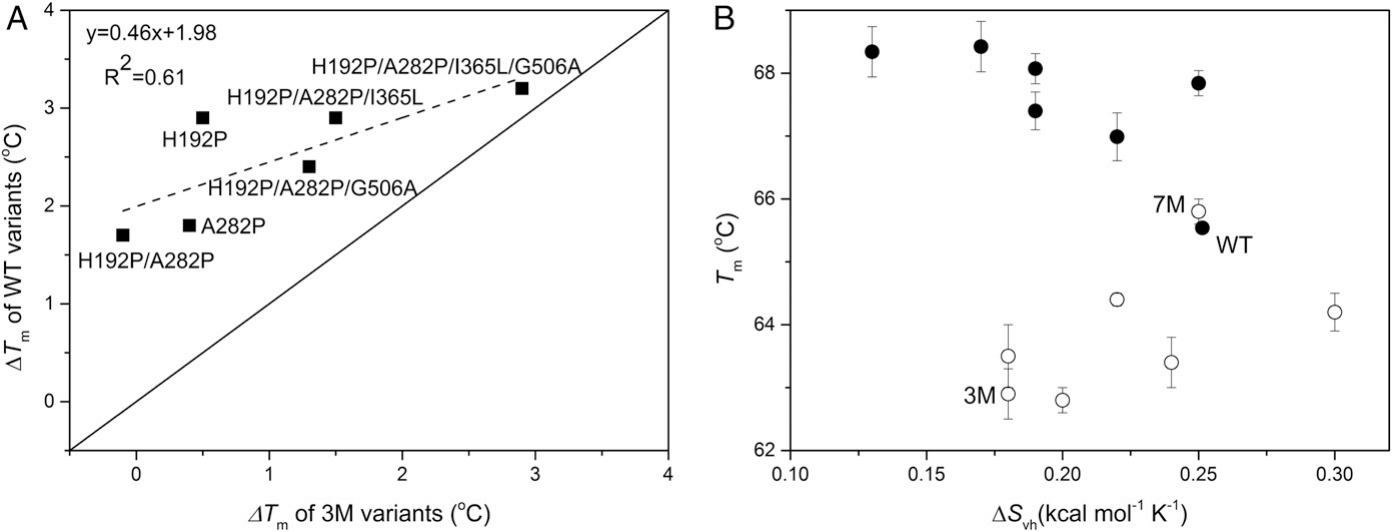
Comparison of the effects of mutations introduced into WT and the 3M variant. (*A*) Effect of mutations on *T*_m_ for the TK variants. The parity line is shown as a solid diagonal. Best line of fit is shown as a dotted line. (*B*) Correlation between *T*_m_ and van’t Hoff entropy for all variants. Variants constructed with WT as the template (●); 3M and variants constructed with 3M as the template (○).

We created six variants—H192P, A282P, H192P/A282P, H192P/A282P/I365L, H192P/A282P/G506A, and H192P/A282P/I365L/G506A—into WT previously (22), and now also into 3M. It was of interest to compare the effects of each variant on stability when introduced into each parent, and these were represented as the differences in *T*_m_ of the variants relative to those of their respective parents (∆*T*_m_). The corresponding ∆*T*_m_ values in each parent had a reasonable correlation (*R*^2^ of 0.61), a slope of 0.5, and an intercept of 2 °C on the WT axis (Fig. 3*A*). This indicated that the effects of each mutation in the two templates were related, but with considerable differences, particularly in the overall magnitudes of effects. The fact that the effects were not identical is consistent with the differences in flexibility between WT and 3M, both at the target sites for mutations and at the active-site/dimer-interface. Previous correlations in ∆*T*_m_ between proteins of similar function and a shared conservation of residues critical to function have been much higher, with a correlation coefficient of 0.82 reported for the impact of mutations on stability when introduced into influenza nucleoprotein homologs of 72% sequence identity (26). In contrast, WT and 3M have different functions, and the three mutated residues in 3M Ser385, Asp469, Arg520 were highly conserved across 52 homologous TK sequences (27). Hence, the specific structural effects of the stabilizing mutations appear to have changed despite the overall high sequence identity, due to the altered patterns of flexibility around the active site in 3M.

A partial correlation between *T*_m_ and *f*_55_ indicated that the cooperativity of unfolding was also changing between the variants, and this was indeed reflected in their ∆*S*_vh_ (Table 2). The WT holo-TK homodimer was found previously to thermally unfold each three-domain monomer, and also dissociate from dimer to monomer, in a single cooperative event (28). Decreased unfolding cooperativity for variants would therefore indicate the decoupled denaturation of at least one structural element from the rest, due to either selective stabilization or destabilization. We examined the correlations between *T*_m_ and ∆*S*_vh_, comparing the variants in 3M, to the equivalent variants in WT (Fig. 3*B*). In previous work, the mutations that stabilized WT improved the *T*_m_ yet also led to lower ∆*S*_vh_ values, indicating a decrease in unfolding cooperativity due to the selective stabilization of one structural feature of the WT TK (22). Meanwhile, the 3M variant engineered previously in WT for altered activity had a lower *T*_m_ but also a lower ∆*S*_vh_, suggesting decreased stability within a structural region affected by the active-site mutations (Table 2). Introducing the same WT-stabilizing mutations into 3M also increased the *T*_m,_ but in contrast to WT they increased ∆*S*_vh_, and hence the unfolding cooperativity (Fig. 3*B* and Table 2). Successive variants improved the unfolding cooperativity of 3M until variant 7M had the same ∆*S*_vh_ as WT (Fig. 3*B*). This difference in behavior indicates that introducing the WT-stabilizing mutations into 3M had specifically restabilized the structural features that were destabilized in 3M. This observation was consistent with our original hypothesis, but further evidence was required to confirm that the mutations restabilized the 3M active-site flexibility via the previously identified dynamically coupled network.

### MD Simulations Analysis of TK Variants.

#### The 3M and WT variants had different number of distinct conformational states.

**Fig. 4.**
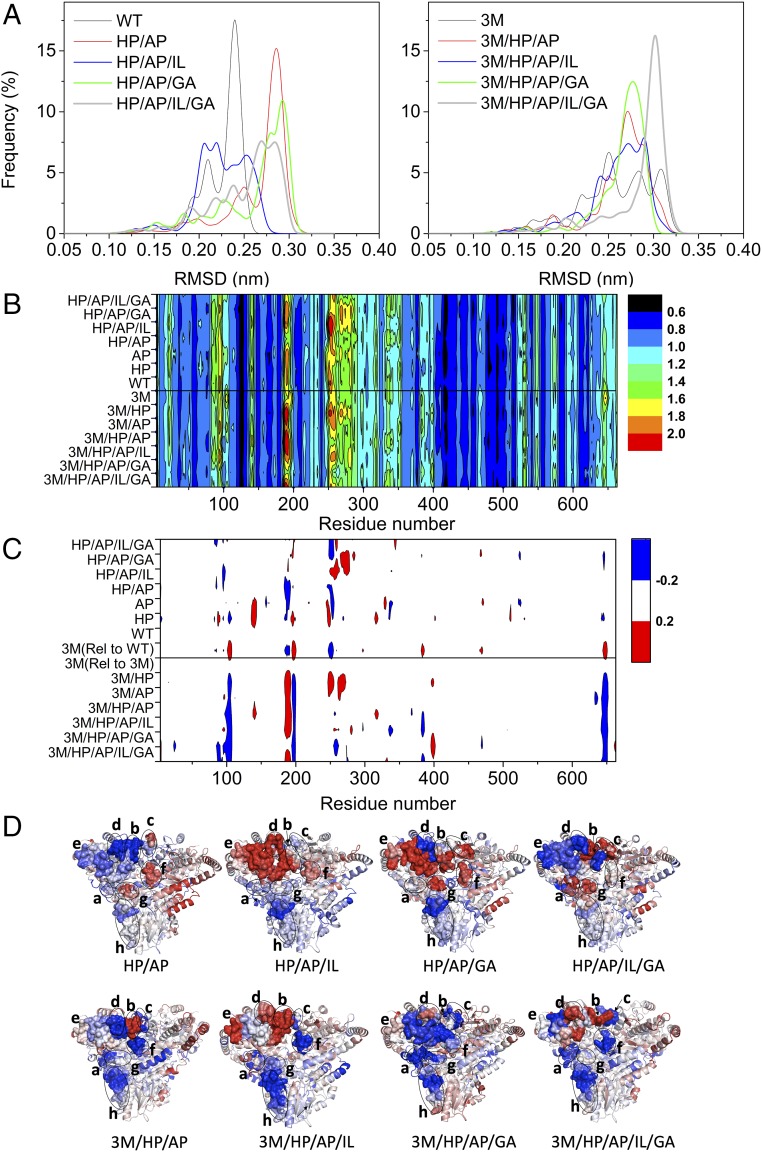
Comparison of flexibility between variants of WT and 3M. (*A*) Distribution of RMSD values from 30-ns simulation trajectories at 370 K. The RMSD values were calculated with the starting structure as the reference. The bin for the distribution is 0.005 nm. (*B*) Heat map indicating the normalized RMSF values of TK variants at 370 K. (*C*) Heat map showing the ΔRMSF of variants relative to their templates, WT (*Upper* half) or 3M (*Lower* half), highlighted red for ΔRMSF > 0.2, blue for ΔRMSF < −0.2, and white for −0.2 < ΔRMSF < 0.2. AP, A282P; GA, G506A; HP, H192P; IL, I365L. (*D*) Structure of variants colored by ΔRMSF relative to WT (*Upper* row) or 3M (*Lower* row), with red indicating high values, and blue for low values. a, G99-Y105; b, N185-H192; c, G195-D200; d, T245-T257; e, H258-A282; f, D381-N386; g, L466-G470; h, V630-K660.

RMSD values calculated for the backbones of all residues were used to explore the overall flexibility of the whole structure (Fig. 4*A*). The distributions of RMSD throughout showed differences in the number of distinct conformational states sampled for each variant. The number of distinct conformational states sampled for 3M was significantly higher than that of WT. The simulation snapshots alignment for WT and 3M also clearly showed that 3M populated distinct alternative conformations at several time points in the simulation, while the WT tended to remain in similar states (Fig. 1*C*). This reflects access to a wider range of distinct structural conformational states for 3M than for WT, and further explains the lower unfolding cooperativity observed for 3M compared with WT, as measured by a significant decrease in ∆*S*_vh_ (Table 2).

WT also showed a minor population of a second conformational state in its RMSD distribution (Fig. 4*A*). Alignment of snapshots from the MD simulation revealed that the movement of the PP-binding domain was more significant compared with other regions including Pyr-binding domain and the C-terminal, particularly the two loops, 245–257 and 185–200, targeted previously for engineering thermostability (*SI Appendix*, Fig. S6) (19). This could lead to an increase in the RMSD values and hence displayed two states of the RMSD distribution. The significant movement of these two loops might also explain why multiple transitions were observed previously for TK during urea denaturation (28). Interestingly, only a single transition was observed in the process of thermal unfolding (*SI Appendix*, Fig. S1). As the second state due to movement of the two loops is at a relatively low population on average, and only one fluorophore, Trp196, is located in these regions, the signal could be relatively silent when measuring the melting temperature based on fluorescence.

Surprisingly, the variants tended to increase the number of states for WT, but decreased the number of states for 3M (Fig. 4*A*). This was consistent with the observation that variants of WT decreased the unfolding cooperativity, and that variants of 3M had higher unfolding cooperativity (Fig. 3*B*). The greater number of conformational states observed in the simulations potentially indicate selective local unfolding or stabilization of at least one structural element that is decoupled from global unfolding of the rest. We therefore analyzed the RMSF for all of the variants, to identify which regions were stabilized or destabilized.

#### Mutations in 3M and WT stabilized different structures within the same correlated network.

To understand which structural features were stabilized or destabilized in the variants of both WT and 3M, relative to the initial structural dynamics and stabilities of their respective parents, we investigated the normalized RMSF values for all variants at 370 K (Fig. 4*B*) and calculated the ΔRMSF between the variants and their respective parents (Fig. 4*C*). The structures of variants are shown in Fig. 4*D*, colored at the residue level by their respective ΔRMSF values. The heat maps showed that the variants did not alter the flexibility significantly in most of the structure (white zones in Fig. 4*C*). Rather, the impact of each mutation in either WT or 3M, increased (red in Fig. 4*C*) or decreased (blue in Fig. 4*C*) the flexibility in a finite number of local structural regions, predominantly D81-K96, G99-Y105, Q136-D143, N185-H192, G195-D200, T245-A282, D381-N386, D469(T), and V630-K660. Apart from D381-N386, found to change only in 3M and its variants, these regions were common to both WT and 3M variants.

Most of the five regions destabilized in 3M (around residues Gly104, Trp196, Ser385, Asp469, and Glu646) became restabilized in the variants of 3M (Fig. 4 *C* and *D*), while additional regions were also stabilized, including D81-K96 and I270-E285 in the 7M variant. Applying the same mutations in 3M and WT confined their impact on flexibility to within the same correlated network, but the specific regions of structure stabilized in WT and in 3M were different (Fig. 4 *C* and *D*). Accordingly, any specific regions of structure that became destabilized were also different between equivalent variants of 3M and WT (Fig. 4*D*). This demonstrates a potential route by which regions outside the active site, but closely coupled through correlation of their dynamics, could provide good targets for stabilizing mutations, even if the target sites were not ranked as the most flexible. This is possible because the mutations can exert their effects across the correlated dynamics network. This effect also explains the partially correlated, but different impacts of applying the same mutations to the two templates WT and 3M (Fig. 3*A*). As the mutations explored here in 3M were previously shown to stabilize WT, it will be interesting to determine in future whether this strategy can be equally successful with unprecedented mutations.

#### Changes in flexibility across the correlated network explain the unfolding cooperativity.

The distribution of flexibility across the correlated network described above can explain the loss of unfolding cooperativity in 3M compared with WT, but also why the same stabilizing mutations then increased the unfolding cooperativity in the 3M variants back to WT levels, and yet decreased it in the variants of WT. Increased flexibility at the dimer interface in 3M would have weakened the interaction between monomers, which could then dissociate the two monomers slightly earlier than monomer unfolding during thermal ramping. This would produce both the observed decrease in *T*_m_ for 3M, and also its lower unfolding cooperativity (Fig. 3*B*).

The mutations in 3M and WT then had different impacts on the flexibilities of each region within the correlated network. For example, the regions around residues 251 and 520 were rigidified by the mutations in WT, but not in 3M (Fig. 4*C*). The flexibility of the loop at T245-T257 and a neighboring region H258-A282, comprising two surface helices plus a loop, were changed dramatically by the mutations in WT (Fig. 4*D*). These regions were more distant from the dimer interface and located on the surface of the PP-domain of TK. The two mutations, H192P and A282P, were located close to this region, and neither mutation on its own could stabilize the T245-T257 loop, leaving part of it more flexible than WT (Fig. 4*C*). However, the combined effect of these two single mutations rigidified the T245-T257 loop (Fig. 4*D*). Subsequent mutations by either I365L or G506A increased the flexibility of H258-A282 to above that in WT, while the quadruple variant, H192P/A282P/I365L/G506A, rigidified it again (Fig. 4 *C* and *D*). Overall, the selective stabilization of the surface region spanning T245-A282 in WT variants would delay monomer unfolding relative to dimer dissociation during thermal ramping, which then explains the gradual increase in *T*_m_ for WT variants, but also their stepwise decreases in unfolding cooperativity (Fig. 3*B*). Variants of WT led to mixed impact on the flexibility at the dimer interface, with some regions stabilized (H94-G108, N185-H192, E642-G649) and others destabilized (G195-D200). Therefore, changes at the dimer interface in WT variants were unlikely to have contributed significantly to changes in *T*_m_ or unfolding cooperativity. This contrasts with the 3M variant itself, for which the dimer interface was much more flexible than in WT.

The five flexible active-site/dimer-interface regions focused around residues 385 and 469 in 3M, were stabilized in the 3M variants, with their flexibility restored to the low levels found in WT (Fig. 4 *C* and *D*). This accounts for the gradual return from low unfolding cooperativity in 3M to the higher unfolding cooperativity in 7M equivalent to that found in WT. Some highly flexible regions in WT were already rigidified in 3M, including T245-T257, W279-I290 targeted previously with the A282P mutation (19), and N185-H192 targeted in WT by the H192P mutation (Fig. 1*B*). These differences explain why the H192P and A282P had little impact initially on the stability of 3M compared with in WT (Table 2). However, the initial H192P and A282P mutations of 3M almost exactly reversed the changes in flexibility that occurred in forming 3M from WT, including restoration of the high flexibility of T245-T257. These effects were balanced out by a simultaneous increase in the flexibility around residues H258-A282, which thus led to no gain in *T*_m_ (Fig. 4*C* and Table 2). The mutations I365L and G506A, leading to variants 5M/G506A and 7M within 3M, were originally targeted as consensus mutations in WT, and yet led back again to lower flexibility in the entire T245-T257 and H258-A282 region (Fig. 4 *C* and *D*). The ability of I365L and G506A to have a stabilizing impact in the 3M variant series was thus facilitated by the earlier restoration of high flexibility at T245-T257, and increased flexibility at H258-A282 in the 3M/H192P/A282P (5M) variant.

## Concluding Remarks

The strategy of RFS has been proven to be a powerful method to improve the stability of enzymes. However, if the highly flexible regions form the active site, or involve key mutations that introduced new functions, modifying them for stability risks losing their activity. We previously found that the dynamics underpinning the flexibility of different regions in a protein were highly correlated across long distances. Specifically, a mutation at one site could influence the dynamics of regions far away from it. Additionally, we have previously used SCA to identify an evolutionarily correlated network that involved active-site residues in TK that we had mutated for function. Directed evolution targeted to that network led to a stabilized a variant with improved soluble expression. We therefore hypothesized here that regions outside the active site whose dynamics were highly correlated to flexible regions within the active site would provide good targets for stabilizing mutations, even if those target sites were not themselves highly flexible.

The 3M variant of TK showed a clear activity–stability trade-off, as well as a loss in unfolding cooperativity indicated by ∆*S*_vh_. MD simulations of 3M showed that this was due to increased flexibility in several interconnected regions at the dimer interface, which also formed part of the active site. The DCCM for 3M identified those regions dynamically correlated with flexibility in the active site. Several mutations found previously to stabilize WT TK were located in these regions, and so to test our hypothesis we introduced the same mutations into 3M. Collectively, the four mutations stabilized 3M via the correlated-dynamics network, and even increased the specific activity by up to threefold toward aromatic substrates. The variants increased progressively both stability and unfolding cooperativity, back to WT levels, where the best variant had a 10.8-fold improved half-life at 55 °C, and increased the *T*_m_ and *T*_agg_ by 3 °C and 4.3 °C, respectively. MD simulations confirmed that the mutations known previously to stabilize the WT, rigidified the dimer interface in 3M and improved the unfolding cooperativity by acting via the correlated network. In future it will be interesting to investigate whether this strategy is equally effective in designing completely unprecedented stabilizing mutations. In summary, this study provides insights into the impact of rigidifying mutations within highly correlated dynamic networks, and also highlights how computational protein engineering strategies might be improved by considering long range dynamics, and also the effects of local dynamics, on the cooperativity of unfolding.

## Materials and Methods

All chemicals were obtained from Sigma-Aldrich, unless mentioned otherwise.

### Site-Directed Mutagenesis, Overexpression, and Purification of Enzymes.

QuikChange XL Site-Directed Mutagenesis Kit (Agilent Technologies) was used to carry out site-directed mutagenesis with TK gene in plasmid pQR791 as the template (28). Primers used for introducing mutation were designed using a web-based QuikChange Primer Design Program (https://www.agilent.com/store/primerDesignProgram.jsp), which were as follows:

**Table:** 

Primer	Sequence
H192P^+^	GGTATTTCTATCGATGGTCCGGTTGAAGGC TGGTTCACC
H192P^−^	GGTGAACCAGCCTTCAACCGGACCATCGATAGAAATACC
A282P^+^	GAACAACTGGGCTGGAAATATCCGCCGTTCGAAATC
A282P^−^	GATTTCGAACGGCGGATATTTCCAGCCCAGTTGTTC
I365L^+^	TAAAGCGTCTCAGAATGCTCTCGAAGCGTTCGGTC
I365L^−^	GACCGAACGCTTCGAGAGCATTCTGAGACGCTTTA
G506A^+^	CGCGTGGAAATACGCTGTTGAGCGTCAGG
G506A^−^	CCTGACGCTCAACAGCGTATTTCCACGCG

Six new mutants produced from S385Y/D469T/R520Q (3M), were S385Y/D469T/R520Q/H192P (3M/H192P); S385Y/D469T/R520Q/A282P (3M/A282P); S385Y/D469T/R520Q/H192P/A282P (5M); S385Y/D469T/R520Q/H192P/A282P/I365L (5M/I365L); S385Y/D469T/R520Q/H192P/A282P/G506A (5M/G506A); S385Y/D469T/R520Q/H192P/A282P/I365L/G506A (7M).

PCR was carried out in a 50-µL reaction system using the standard protocol in the QuikChange manual. 3M/H192P and 3M/A282P were constructed with the gene encoding S385Y/D469T/R520Q as the template while 5M was constructed using A282P primers with 3M/H192P as the template. With the gene of 5M as the template, we constructed the 5M/I365L and 5M/G506A. 7M was constructed using G506A primers with gene of 5M/I365L as the template. The PCR products after digestion of the template by DpnI, were transformed into XL10-Gold ultracompetent cells contained in the kit. TK mutants were expressed, purified, and quantified as previously reported (19).

### Enzyme Activity Measurement.

With Li-HPA as the donor substrate, WT TK and its variants were tested for reactions with four acceptor substrates GA, PA, 3-FBA, and 4-FBA. All reactions were run in triplicate at 22 °C. For the substrates of GA and PA, reactions were initiated by adding 50 µL of 150 mM Li-HPA and 150 mM GA or PA in 50 mM Tris⋅HCl, pH 7.0 into 100 µL enzymes with concentration of 0.1 mg/mL Then, the reactions were quenched at various time over 60 min for GA and 24 h for PA, by adding 10 µL sample into 190 µ L 0.1% (vol/vol) TFA, before determination by HPLC with an aminex HPx-87H, 300 × 7.8-mm column (Bio-Rad). For the substrates of 3-FBA and 4-FBA, reactions were initiated by adding 100 µL of 150 mM Li-HPA and 150 mM 3-FBA or 4-FBA in 50 mM Tris⋅HCl, pH 7.0 into 200 µL enzymes with concentration of 0.1 mg/mL The reactions were quenched at various time over 2 h for 3-FBA and 24 h for 4-FBA, by adding a 20-µL sample into 380 µL 0.1% (vol/vol), before determination by HPLC with an ACE5 C18 reverse phase column (150 × 4.6 mm).

### Temperature Inactivation of Holo-TK.

Thermostability was assessed as the activity retained after exposure to high temperatures. Enzyme activity was tested before and after heating at 55 °C for 1 h or 60 °C for 10 min, with 3-FBA and Li-HPA as the substrates. Retained activity was calculated by dividing the activity of heated enzymes by their initial activities. The half-life of enzyme activity was measured in triplicate by placing 100-µL enzymes (0.1 mg/mL) at 55 °C in a thermocycler. Samples were removed at different times and then cooled to 25 °C. Enzyme activity was measured as above. As described previously (22), a second-order deactivation rate constant (*k*_d_) was measured by linear regression of 1/(retained activity) versus the incubation time (*t*). The half-life (*t*_1/2_) of each variant at 55 °C was calculated using Eq. **1**.

$$t_{1/2}=1/\left(100*k_{d}\right)$$ [1]

### Enzyme Kinetics.

Kinetic parameters were obtained at saturating 50 mM Li-HPA levels and a range of 0.5–40 mM 3-FBA in final conditions of 50 mM Tris⋅HCl, 2.4 mM ThDP, 9 mM MgCl_2_, pH 7.0. The mixtures containing enzymes (0.067 mg/mL) and substrates were incubated at 22 °C for 15 h. Each 20-µL reaction sample was taken at 1, 3, 5, 10, and 15 min and quenched by adding 380 µL of 0.1% (vol/vol) TFA. Triplicate reactions were monitored using HPLC as above. All data were fitted by nonlinear regression to the Michaelis–Menten equation to determine the *K*_m_ and *k*_cat_ of WT TK and the variants using software OriginPro9.0. Double-reciprocal Lineweaver–Burk plots were also used to verify that the relationships between the velocity and the substrate concentration were linear.

### *T*_m_ and *T*_agg_ Temperatures.

Intrinsic protein fluorescence (266-nm excitation, 280 to 450-nm emission scan) and SLS at 266 nm and 473 nm, were measured simultaneously for measuring the *T*_m_ and *T*_agg_ values of TK variants using a UNit (Unchained Laboratories). The detailed procedures and data analysis were carried out as reported previously (22), and as also found in *SI Appendix*.

### MD Simulations.

The RING 2.0 web server was applied for predicting residue interaction networks (29). MD simulation software Gromacs v5.0 was used to investigate the structural flexibility of the TK WT (PDB ID code 1QGD), the variant S385Y/D469T/R520Q (PDB ID code 5HHT), and variants constructed with the Pymol Mutagenesis Wizard (Schrödinger). Simulations were carried out using the OPLS-AA force field. The initial structure was solvated in a cubic simulation box with a layer of water at least 10.0 Å from the protein surface. Sufficient Na^+^ was added to neutralize the negative charges in the system. The whole system was minimized using the steepest descent method (2,000 steps) plus the conjugate gradient method (5,000 steps). Two 50-ps position-restricted simulations were performed under NVT and NPT ensembles, respectively, with heavy atoms and C_α_-atoms fixed. Finally, a 30-ns MD simulation was performed in triplicate on the whole system at 300 K and 370 K. RMSDs were calculated for the group of backbone using initial structure as the reference. RMSFs of the whole protein calculated using the last 10-ns trajectory were applied for analysis of local flexibility of the proteins. The DCCM was calculated as reported previously (22).

## Supplementary Material

Supplementary File
