# Supplementary material for: Exploiting correlated molecular-dynamics networks to counteract enzyme activity–stability trade-off
Source: Proc Natl Acad Sci U S A. 2018 Dec 10;115(52):E12192–200. doi: 10.1073/pnas.1812204115 (PMC6310800; doi:10.1073/pnas.1812204115)
Supplement: Supplementary File [file pnas.1812204115.sapp.pdf]

PNAS
www.pnas.org

Supplementary Information for

Exploiting correlated molecular-dynamics networks to counteract enzyme activity-stability trade-off

Haoran Yu, Paul A. Dalby*

Correspondence and requests for materials should be addressed to P. A. D.
Email: p.dalby@ucl.ac.uk

This PDF file includes:

Supplementary text
Figs. S1 to S8
References for SI reference citations

www.pnas.org/cgi/doi/10.1073/pnas.1812204115

Methods

Interactions network predictions. The RING 2.0 web server was applied for predicting residue interaction networks (1). Wild-type (1QGD.pdb) and 3M variant (5HHT.pdb) structures were used as the input. The distance thresholds were set as the option of Relaxed, which corresponds to the distance thresholds including hydrogen bond (5.5 Å), salt bridge (5.0 Å) disulphide bond (3.0 Å), Van-der-Waals (0.8 Å) and π- π stacking (7.0 Å). During the calculation, the network policy was set as the Closest and the interactions with water and hetero atoms were not considered. Only the most probable interaction type was calculated between a residue pair. The output files were imported to Cytoscape for further analysis (2) .

Thermal transition mid-point (T_m) and aggregation onset (T_{agg}) temperatures. Intrinsic protein fluorescence (IPF) (266 nm excitation, 280-450 nm emission scan) and static light scattering (SLS) at 266 nm and 473 nm, were measured simultaneously for measuring the T_m and T_{agg} values of TK variants using a UNit (Unchained Laboratories, Pleasanton CA). Measures were taken as a function of temperature in the range of 30-90 °C with steps of 1 °C, equilibration time of 30 s at each temperature. The microcuvette arrays were loaded with 9 µL of 0.1 mg/mL sample and all experiments were performed in triplicates. T_{agg} values were determined with instrument software by the analysis of SLS counts at 266 nm. Fluorescence intensity ratio determined between the spectral intensity at 350 nm to that at 330 nm was used to calculate melting temperature, $T_{m.}$ The fluorescence intensity ratio was fitted to a two-state transition model using equation 2 (3, 4), in the software OriginPro 9.0 (Origin Lab Corp., Northampton, MA, USA).

$$I_T = \frac{I_N + aT + (I_D + bT)\exp\left[\left(\frac{\Delta H_{vh}}{R}\right)\left(\frac{1}{T_m} - \frac{1}{T}\right)\right]}{1 + \exp\left[\left(\frac{\Delta H_{vh}}{R}\right)\left(\frac{1}{T_m} - \frac{1}{T}\right)\right]} \qquad \text{Eq. 2}$$

where I_T is the observed signal, I_N and I_D are the native and denatured baseline intercepts, a and b are the native and denatured baseline slopes, T is the temperature, ΔH_{vh} is the van't Hoff enthalpy, R is the gas constant (1.987 cal mol^{-1} K^{-1}) and T_m is the apparent mid-point of the observed thermal transition. The van't Hoff entropy is calculated using equation 3.

$$\Delta S_{vh} = \frac{\Delta H_{vh}}{T_m} \qquad \text{Eq. 3}$$

The mole-fraction, f_T, of unfolded protein at any temperature T, was calculated from

$$f_T = \frac{I_T - I_N - aT}{I_D + bT - I_N - aT} \qquad \text{Eq. 4}$$

and by substituting for I_T in the equation 2, this reduces to

$$f_T = \frac{\exp\left[\left(\frac{\Delta H_{vh}}{R}\right)\left(\frac{1}{T_m} - \frac{1}{T}\right)\right]}{1 + \exp\left[\left(\frac{\Delta H_{vh}}{R}\right)\left(\frac{1}{T_m} - \frac{1}{T}\right)\right]} \qquad \text{Eq. 5}$$

Results

Why did 5M/I365L have the highest kinetic stability and T_{agg}, but modestly increased T_m? It is not easy to explain why 5M/I365L had the longest half-life at 55 °C, whereas its Tm was not the highest among the variants (Table 2). Some differences between 5M/I365L and the other variants were observed, and these may have contributed in some way to its outlier behaviour. First, 5M/I365L decreased the flexibility at G331-K347, which was not found for other variants of 3M (Fig. 4 C & S7). The G331-K347 fragment was in close proximity to the mutation I365L (Fig. S8). It was also one of the most flexible regions in the TK Pyr-domain (323-539 aa). The flexibility of the whole Pyr-domain for 5M/I365L was lower on average than for other variants, which may have been critically linked to stabilization at G331-K347 (Fig. S7). On the other hand, in the PP-domain (2-322 aa) of the 5M/I365L, several regions around residues 94, 192, 254 and 282 became more flexible than in 3M and other variants (Fig. S7). Unlike other variants, 5M/I365L had a relatively more stable Pyr-domain and a less stable PP-domain compared to other variants including 7M and 5M/G506A.

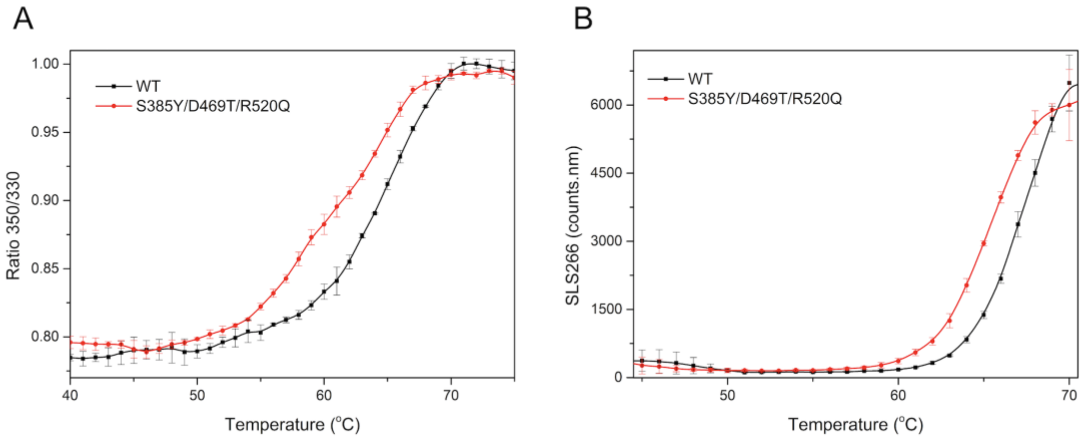

Fig. S1. Thermal stability of TK WT and the 3M variant. A, thermal transition mid-point (T_m) determination by intrinsic fluorescence. B, aggregation onset temperature (T_{agg}) determination by static light scattering.

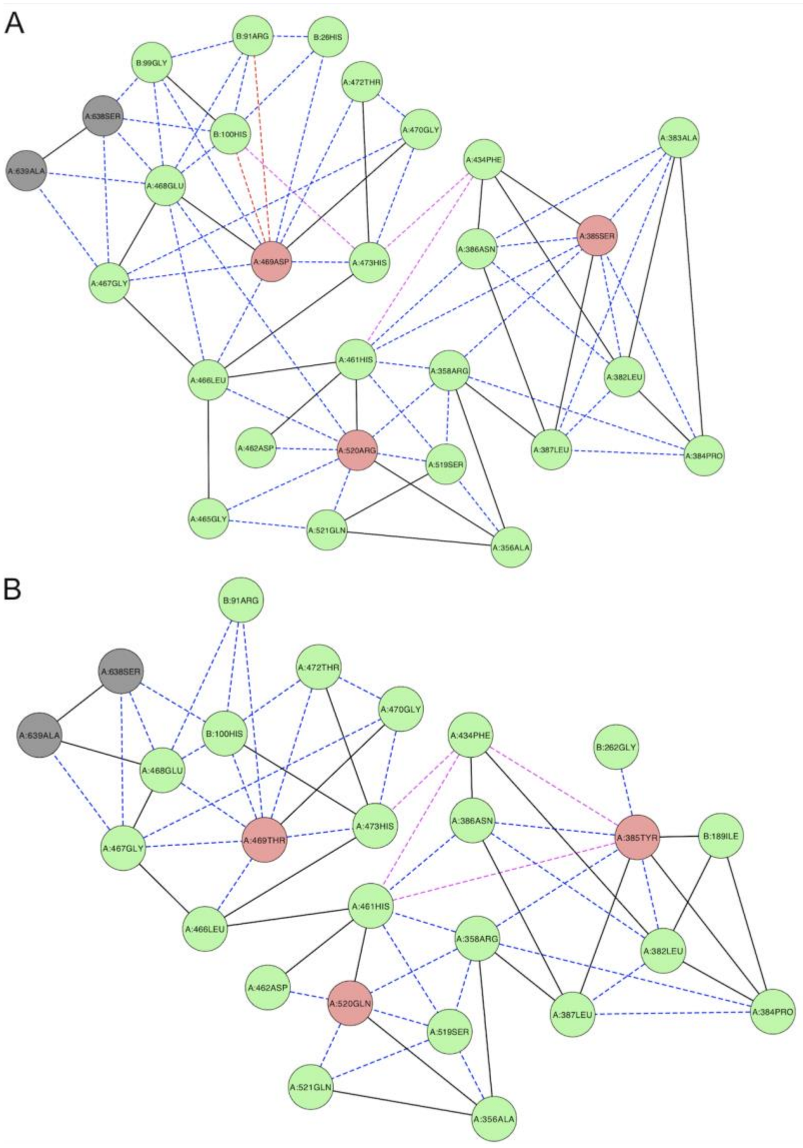

Fig. S2. Residue interaction networks of the local region around the 3M mutation sites for WT and 3M. A, Interaction network for WT calculated using 1QGD.pdb. B, Interaction network for 3M calculated using 5HHT.pdb. Nodes corresponding to residues are coloured by red (mutation sites), green (residues directly connecting with at least one mutation site), grey (residues indirectly connecting with mutation sites). Edges corresponding to non-covalent interactions are coloured as solid black lines (Van der Waals), blue dashed lines (hydrogen bonds), red dashed lines (salt bridges), pink dashed lines (π-π stacking).

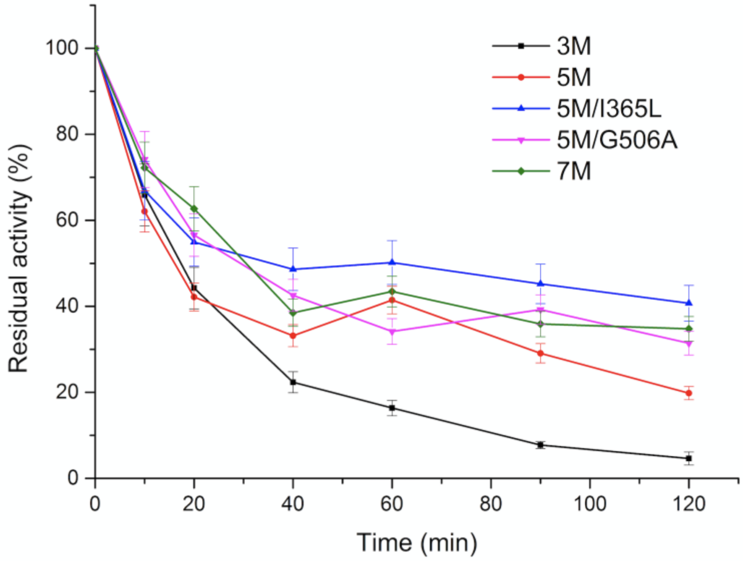

Fig. S3. Inactivation profiles of TK variants at 55 °C. Enzymes in 100 µL solution (2.4 mM TPP, 9 mM $MgCl_2$ and 50 mM Tris-HCl, pH 7.0) were incubated at 55 °C for different times and assayed for residual activity at 22 °C.

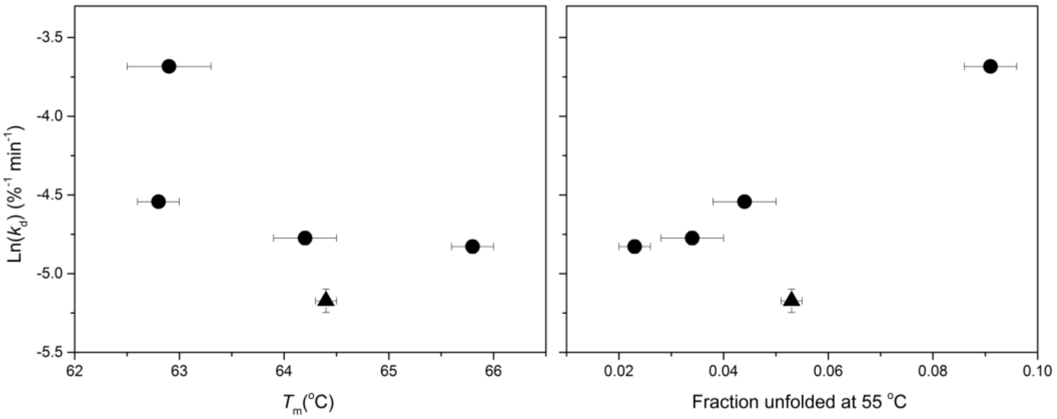

Fig. S4. Correlation between T_m or f_{55} and deactivation rate constant for TK variants. (●) 3M, 5M, 5M/G506A, 7M; (▲) 5M/I365L

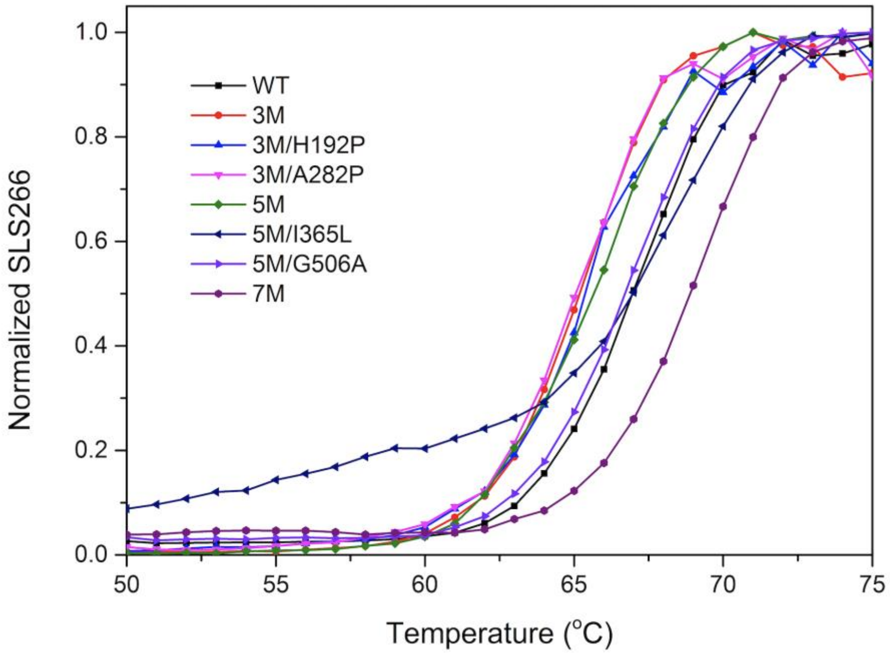

Fig. S5. Thermal aggregation profiles detected by static light scattering for the WT and variants. The SLS266 data were normalized for comparison.

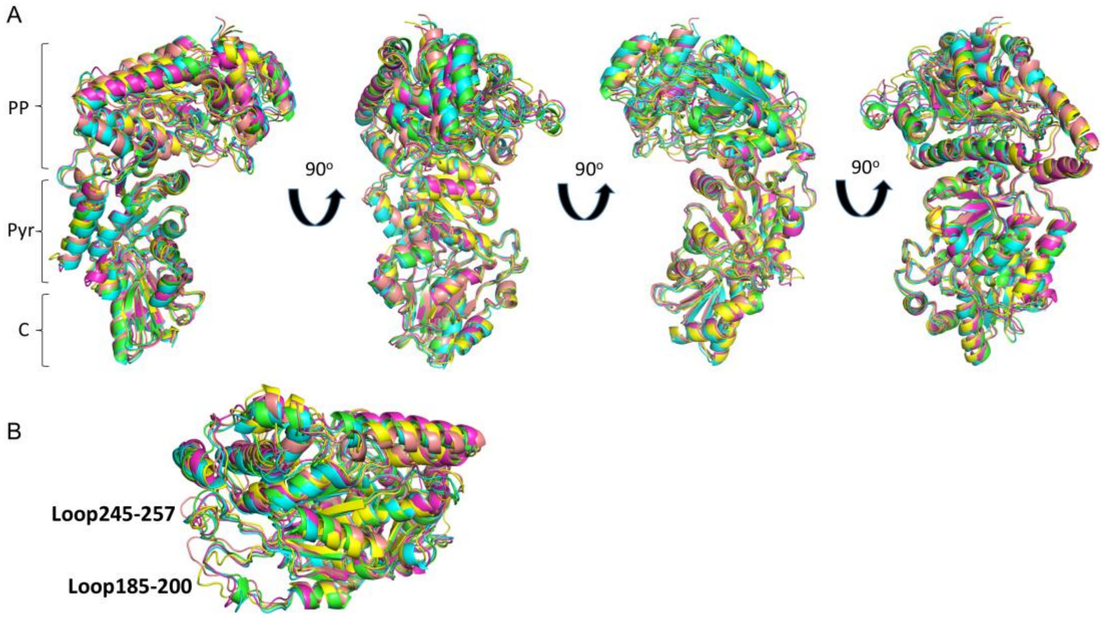

Fig. S6. Alignment of snapshots from the MD simulation of WT at 370 K. A, Alignment of single chain structure at different time points. PP, phosphate binding domain; Pyr, pyridine binding domain; C, C-terminal. B, top view of PP-binding domain in the structures alignment. green, 5 ns; cyan, 7.5 ns; purple, 10 ns; yellow, 20 ns; pink, 30 ns

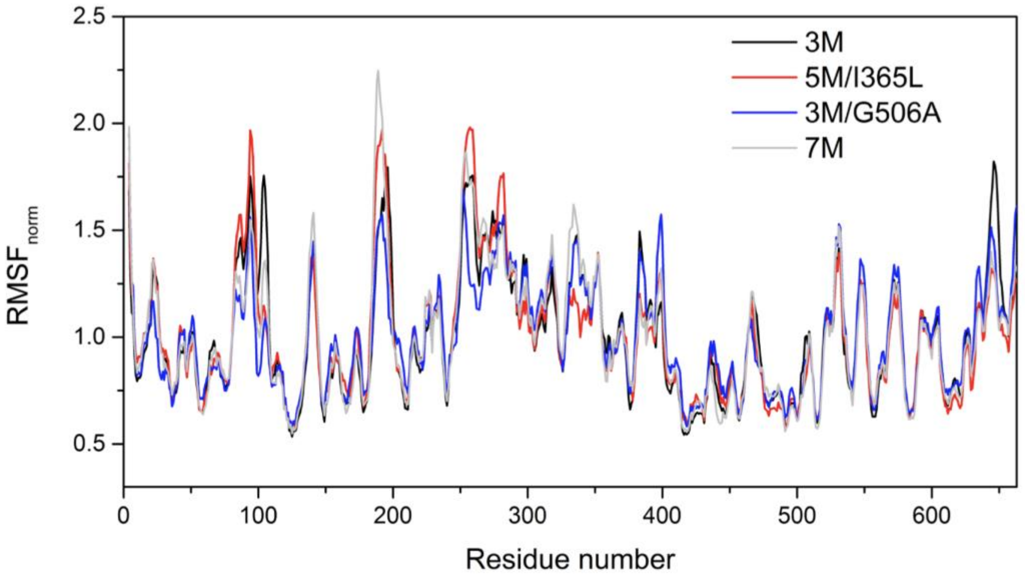

Fig. S7. Normalized RMSF of TK variants at 370 K. The data shown were the average of triplicate simulation trajectories.

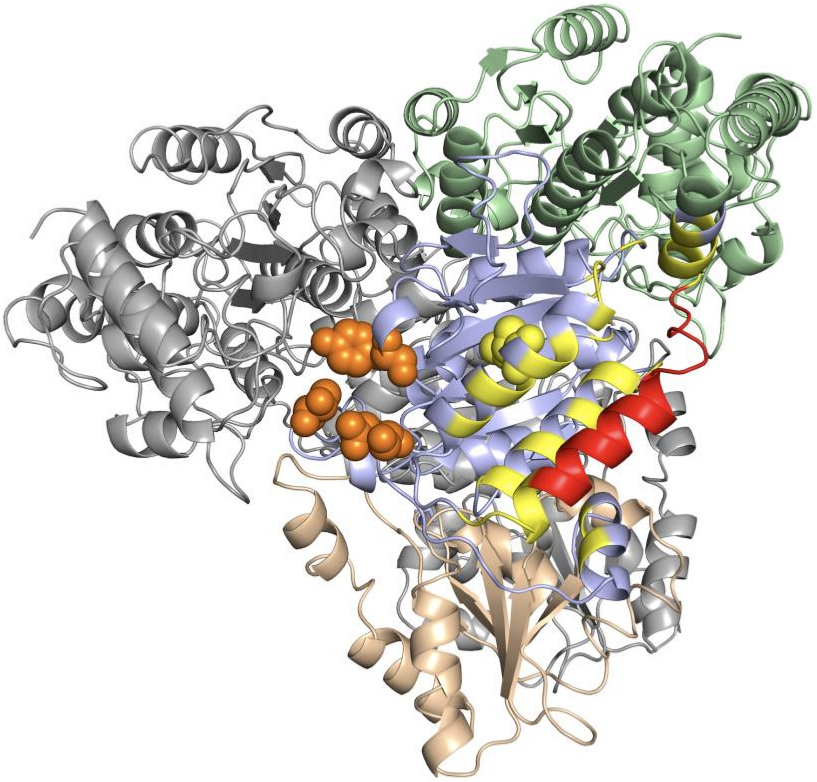

Fig. S8. Structure of 3M variant (5HHT.pdb). Red, a fragment Gly331-Lys347 in chain A; Yellow, the residues around fragment Gly331-Lys347 within 6 Å; Green, PP-domain of chain A; Blue, Pyr-domain of chain A; Wheat, C-terminal of chain A; Grey, chain B of TK. Three mutations S385Y, D469T, R520Q were shown as orange spheres. I365L were shown as a yellow sphere.

References

1. Piovesan D, Minervini G, & Tosatto SC (2016) The RING 2.0 web server for high quality residue interaction networks. *Nucleic Acids Res* 44(W1):W367-374.
2. Shannon P*, et al.* (2003) Cytoscape: a software environment for integrated models of biomolecular interaction networks. *Genome Res* 13(11):2498-2504.
3. Santoro MM & Bolen DW (1988) Unfolding free energy changes determined by the linear extrapolation method. 1. Unfolding of phenylmethanesulfonyl alpha-chymotrypsin using different denaturants. *Biochemistry* 27(21):8063-8068.
4. Consalvi V*, et al.* (2000) Thermal unfolding and conformational stability of the recombinant domain II of glutamate dehydrogenase from the hyperthermophile Thermotoga maritima. *Protein Eng* 13(7):501-507.
